# Prevalence of non-odontogenic infectious lesions of oral mucosa in a group of Iranian patients during 11 years: a cross sectional study

**Published:** 2019-10

**Authors:** Sara Haghighat, Fahimeh Rezazadeh

**Affiliations:** 1 Department of Oral and Maxillofacial Medicine, Student Research Committee, School of Dentistry, Shiraz University of Medical Sciences, Shiraz, Iran; 2 Department of Oral and Maxillofacial Medicine, Oral and Dental Diseases Research Center, School of Dentistry, Shiraz University of Medical Sciences, Shiraz, Iran

**Keywords:** Epidemiology, Infectious disease, Oral lesion, Mucosal lesion, Fungal disorder

## Abstract

**Background and Objectives:**

Oral mucosal infections are an important type of oral lesions. The aim of this study was to determine the epidemiology of oral mucosal infectious lesions in patients who referred to Oral Medicine Department of Shiraz Dental School, Iran during 11 years.

**Materials and Methods:**

In this cross sectional study, records of all patients who referred to Oral Medicine Department of Shiraz Dental School from September 2007 to January 2018 were assessed and those data sheets which their definitive diagnosis were a kind of oral mucosal infectious lesion were recorded. Pearson Chi-square test was used for statistical analysis. Level of significance was considered as P value < 0.05.

**Results:**

Overall prevalence of oral mucosal infectious lesions was 9.47%. Generally, mean age of patients was 42.92 ± 18.84 and most of them were female. Most common type of infectious lesions was fungal infections, but viral and bacterial infections were less common. Among fungal infections, most lesions were candidiasis and only 3 cases were diagnosed as deep fungal infection. HSV infection was the second common oral infectious lesion. There was a significant relation between infectious lesion and systemic disease or medication use (P=0.000).

**Conclusion:**

This study is the first epidemiologic study in Iran, concerning oral mucosal infectious lesions. Total of 9.47% of oral lesions were infective, candidiasis and HSV lesions were the most common oral mucosal infective disease, which were more prevalent amongst female, middle age people and patients with systemic disease.

## INTRODUCTION

Oral mucosal infection is an important type of oral lesions. The oral mucosa has as a protective function against pathogens and chemicals that enter the oral cavity; thus, infectious lesions in this area could significantly affect the treatment plans and prognosis of routine dental practice. In recent years, due to lack of sufficient information on mucosal lesions, there has been a growing emphasis on the epidemiologic studies about oral mucosal disorders (1). The results of these studies can lead to timely primary prevention, early diagnosis, and prompt treatment.

Oral mucosal infections can be divided into fungal, viral and bacterial lesions. Since 1980, fungal infections are known as an important public health problem, and their diagnosis and treatment of such diseases are substantial. For instance, oral candidiasis is a kind of opportunistic fungal infection, known as one of the major cause of human diseases, especially in immunocompromised patients (2). It is mostly produced by *Candida albicans* being the most important from public health point of view, while other species such as *C. glabrata, C. parapsilosis, C. tropicalis, C. krusei* and more recently *C. dubliniensis, C. colliculosa, C. fomata, C. guillermondii* and *C. rugosa* have been also isolated (3, 4). Despite its great importance as an opportunistic infection, there is little known about the prevalence of oral candidiasis in different geographic regions. Deep fungal infections are a worldwide health issue, since they are life threatening; hence, their early diagnosis and treatment is crucial (5).

Viral infections are another type of oral infectious lesions which can develop as a result of the activity of viruses such as Human Papilloma Virus (HPV), Herpes Simplex Virus (HSV), etc. During the past two decades, numerous studies have associated HPV infection as an important causal factor of epithelial neoplasm, such as head and neck Squamous Cell Carcinoma (SCC) and oropharyngeal cancer (6–9). It has also been suggested that HSV might have active role in initiating oral SCC (10–12) and Pemphigus Vulgaris (13).

Oral bacterial infection due to dental or periodontal problem is a common disease. However, little information is available regarding the origins of bacterial infection. Despite improvements in diagnosis and treatment of dental caries, there are still challenges in dealing with consequences of untreated caries such as odontogenic infections, especially in the developing countries. During the last decade, there has been a notable increase in the severity of odontogenic infections (14). Some of which might be undiagnosed or untreated by being expressed as a chronic soft tissue swelling. It is important to study about bacterial mucosal infections such as sialadenitis, cellulitis and infected cysts. These lesions are important because their misdiagnosis can result in treatment failure and chronicity.

In previous studies in term of oral lesions only candidiasis was mainly investigated. Since there are few studies that have documented the entire range of possible infectious oral lesions in the population, this cross-sectional study was done in Oral Medicine Department of Shiraz Dental School, a main center for oral diseases in Southwestern Iran. The aim of this study was to determine the epidemiology of oral mucosal infectious lesions in patients who referred to Oral Medicine Department of Shiraz Dental School, Iran during 11 years (September 2007–February 2018).

## MATERIALS AND METHODS

In this cross sectional study, records of all patients who referred to Oral Medicine Department of Shiraz Dental School from September 2007 to January 2018 were assessed and those data sheets which their definitive diagnosis were a type of oral mucosal infectious lesion were recorded. Our research was approved by local Ethics Committee of Shiraz University of Medical Sciences (ethics code=IR.SUMS. REC.1397.959). In this department, only patients with oral lesions other than routine dental problem after clinical exam by probe and radiography (caries, dental pain, periodontal disease) and referral patients with an oral and maxillofacial problem were assessed. Each lesion was assessed clinically by oral medicine specialist and if necessary, complementary examination was made (including laboratory tests, aspiration, radiographic and histopathologic evaluation and in some cases more complicated experimental methods such as cultivation and PCR analysis). Following confirmation of the diagnosis, appropriate treatment was done. In this study, the patients data were collected from their clinical examination (lesion description), history, the underlying etiology, and final diagnosis. Oral mucosal infectious lesions were divided into four categories: (I) fungal infection, (II) viral infection, (III) unusual bacterial infection, and (IV) other causes. In present study common dental bacterial infections (acute abscess or cellulitis) were not included because these were easily diagnosed by general dentist or in dental diagnosis department. Chronic undiagnosed dental infection or those with uncommon feature were mostly referred to oral medicine department.

Records with incomplete data in data sheets, absence of definitive diagnosis or lack of patient follow up were excluded. Data included age, gender, type of infectious disease, detection date (year), type of systemic disease, use of any medication, smoker or not and socioeconomic status (level of education, job, and place of living) that were analyzed by SPSS 17 software. Pearson Chi-square test was used to assess the relationship between different variables. P value <0.05 (α=0.05) at a 95% confidence interval (C.I.) was considered to be statistically significant.

## RESULTS

**Fig. 1:**
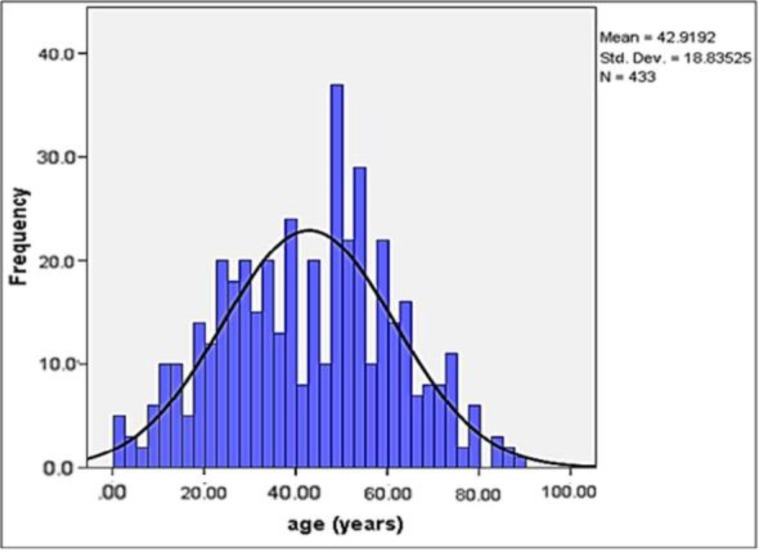
Age of patients in the study

Among the 4676 data sheets, a total of 443 cases were diagnosed with oral mucosal infectious lesions. Overall prevalence of oral mucosal infectious lesions was 9.47%. Most of the patients had referred during autumn season (37%). Generally, patients mean age was 42.92 ± 18.84 and majority of them were female (59.1%). (Fig. 1 and Table 2).

Table 1 shows the general clinical characteristics. The oral lesions identified were classified as (I) fungal, (II) viral, and (III) bacterial infections. Most common type of infectious lesion was fungal infections (38.6%). Viral and bacterial infections were less common with 31.8% and 25.5% occurrence, respectively. Among fungal infections, most of the lesions were candidiasis (n=168, 37.9%) and only 3 cases were diagnosed as deep fungal infection. HSV infection was the second common oral infectious lesion with a prevalence of 19.9% (Table 1).

**Table 1:** Subtypes of infectious lesions pathogens

**Name of pathogen**	**Frequency**	**Percentage**
Candidiasis	168	37.9
Deep fungal infections	3	0.7
HSV	88	19.9
HPV	34	7.7
Other viral infections (VZV, CMV, EBV)	13	2.9
Dental abscess	23	5.2
Infected cyst	36	8.1
Sialadenitis	15	3.4
Other bacterial infections (sinusitis/cellulitis)	29	6.5
Other infections (parasite/trauma/infected ulcer)	33	7.4

Health conditions of patients (presence of any systemic disease such as cardiovascular or immunodeficiency disorders and using any kind of medication) were also recorded. (Table 2) Using Chi-square test, there was a significant relationship between infectious lesions and systemic disease or medication use (P=0.000).

**Table 2:** Age, gender and health conditions of patients in the study

	**Mean Age**	**Gender**		**Systemic Disease**		**Use Of Medication**	
			
	**(±S.D.)**	**Male**	**Female**	**Presence**	**Absence**	**Yes**	**No**
Fungal	51.12 (±16.79)	67 (39.2%)	104 (60.8%)	117 (68.4%)	54 (31.6%)	105 (62.1%)	64 (37.9%)
Bacterial	37.61 (±16.51)	51 (45.1%)	62 (54.9%)	33 (29.2%)	80 (70.8%)	37 (33.0%)	75 (67.0%)
Viral	36.29 (±19.10)	54 (38.3%)	87 (61.7%)	48 (34.3%)	92 (65.7%)	56 (40.6%)	82 (59.4%)
Other (Parasite)	49.33 (±18.19)	9 (50%)	9 (50%)	9 (50.0%)	9 (50.0%)	11 (61.1%)	7 (38.9%)
Total	42.92 (±18.84)	181 (40.9%)	262 (59.1%)	207 (46.8%)	235 (53.2%)	209 (47.8)	228 (52.2)

**Fig. 2:**
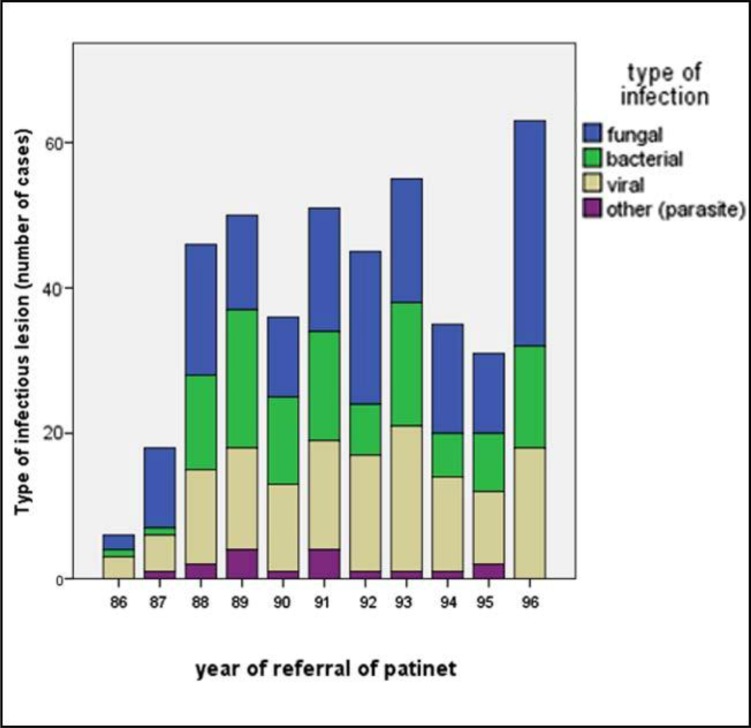
Prevalence of infectious lesions during 11 years (1386 to 1396)

Socioeconomic status of patients in the study is shown in Table 2. Most patients had high school diploma or higher and mostly were residing in Shiraz. There was no any correlation between socioeconomic status and prevalence of lesions (P =0.009). Fig. 2 reports the prevalence of oral infectious lesions within the past 11 years (the trend of lesions occurrence during time) and the last year had the highest prevalence, showing a gradual increase in their occurrence.

## DISCUSSION

Epidemiologic studies usually provide valuable data about the contributing factors, incidence and distribution of disorders in a group of people. These studies help to identify the disease process and cause of affecting various groups of patients (14). Epidemiology of oral infectious diseases is important to understand the public health impact by evaluating methods of controlling these diseases.

The diagnosis of the different oral lesions is an essential part of oral health care services and their prevalence is an important parameter of the oral health. One group lesions is oral mucosal infectious lesions that WHO stated that after dental and periodontal diseases, it is one of the most common oral diseases. Different type of lesions with various etiology and risk factors resulted in complexity of our study. In this research we examined 3 main group of lesions; fungal, viral and bacterial group.

Before discussing the results of our study, it is essential to explain the setting of Oral Medicine Department of Shiraz Dental School. It is a department that patients with chief complaints other than only dental caries or periodontal problems are referred. Diagnosis and treatment is done by oral medicine residents and is confirmed by oral medicine specialists, which might have influenced our results.

The overall prevalence of oral mucosal infectious lesions was 9.47% between all of oral mucosal lesions. Our findings indicated that the most prevalent infectious lesion was oral candidiasis (37.9% of all infectious cases and 3.59% of all mucosal lesions). To the best of our knowledge, there was no similar study in Iran that evaluated oral infectious lesions. Although other epidemiologic studies that focus on oral lesions, might have pointed to some lesions (16–19). Ghanaei et al. investigated oral mucosal lesions in Rasht, Iran in 2013 and reported a prevalence of 6.9% of candidiasis among all diagnosed mucosal lesions (16). There were also studies done in Lebanon, Turkey, Italy and Mexico which reported the prevalence of candidiasis at 2.2%, 1.4%, 5.5% and 1.9%, respectively (1, 17–19). All of these studies assessed the prevalence of oral mucosal lesions based on clinical feature; however, in our study only infectious lesions were evaluated. Hence, in these studies different low prevalence rates were reported.

HSV was the second prevalent lesion with 19.9% among all cases in our study. Herpes labialis had 5.3% prevalence in the study conducted by Ghanaei et al. (16) and 11%, 13.14% and 0.97% in other studies (1, 17, 19). In Cebeci et al. and Amadori et al. studies HSV was the most prevalent infectious oral mucosal lesion that differed from our results (1, 19). This might have been due to different sample group or diagnostic methods.

Infected cysts and HPV are the third and fourth prevalent lesions considering 8.1% and 7.7% of all the lesions. Infected cysts are formed mostly because of long lasting extended dental infections and caries. HPV had less prevalence than candidiasis and HSV in other studies, which is compatible with our results. Although not frequent, there was 3 cases with deep fungal infection (mucoromycosis) that timely diagnosis and treatment of them is extremely important and delay in treatment of such infections could be life threatening.

Our study showed that number of diagnosed infectious lesions has an overall increasing trend during 11 years (Fig. 2). This probably is due to increase in number of overall referred patients and their knowledge and attitude about their oral lesions. Improvements in the diagnostic methods including clinical assessment and histopathologic methods might have also led to this increase.

Health conditions of patients in the study were assessed, and presence of any systemic diseases using medications was evaluated. Overall, patients without any systemic diseases were significantly more than those with a kind of systemic disorder, but taking a more specific look at the results shows that this relationship is not evident in fungal infections. Total of 68.4% of patients diagnosed with fungal infections had a systemic disease, indicating the importance of host susceptibility in being affected by oppurtinstic fungi-most commonly candidiasis (4, 5).

Moreover, most of the patients did not use any kind of medications. Again in fungal infections there is a different sceniario. 62.1% of patients diagnosed with fungal infection used a kind of medication that confirms the greater number of patients with a systemic disease.

In this study, lesions were more in females (59%) than males, which was not in line with Bhatnagar et al. (15), Rajendra et al. (14) and Mansour Ghanaei et al. (16) results; however, it was in accordance with the study done by Castellanos et al. 2008 (17). This might be due to the higher number of patients reffered or the higher prevalence of fungal infections in females (16). Hence, some conditions are highly influenced by gender.

The prevalence of oral mucosal infectious lesions were more in middle aged patients (mean age = 42.92 ± 18.84). This finding is almost similar to several other studies (16, 17, 19). This could be related to the most common lesion, candidiasis which is more common amongst the elderly; however, The prevalence of viral infections, especially primary HSV lesions were higher in children.

Prevalence of infective diseases were significantly different, cosidering socioeconomic status of patients. There was a significant relationship between the pravalence and level of education, and also place of residence. Most of the patients had high school diploma or higher and lived in Shiraz (Table 3). This might be due to paying more attention to health; thus, requesting more professional dental care in more educated people. Availability of dental clinics was also another factor that caused most of the patients to referr to Shiraz, capital of Fars province in comparison with other cities.

**Table 3:** Socioeconomic status of patients in the study

**Variables**	**Percentage of patients with infectious lesions**
**Level of education**	
no education	26.1%
under diploma	26.5%
diploma or higher	47.4%
**Occupation**	
with job	39.5%
without job	60.5%
**Place of residence**	
Shiraz	62.9%
Cities around Shiraz (Fars province)	37.1%

This study was done to encourage dentists to examine oral lesions correctly and for oral health managers to evaluate the prevalence of oral lesions using standard methods. The present study has some limitations which related to the type of study (cross sectional) and also some incompleted patient’s file or data. So further studies with larger population, in multiple centers with more precise technique for lesions evaluation is suggested.

## CONCLUSION

This study is an epidemiologic study in Iran that specifically evaluated oral mucosal infectious lesions. It provides information about the epidemiologic aspects of this group of oral lesions that might perovide valuable data in planning for the oral health services. In total, 9.47% of oral lesions were infective, candidiasis, and HSV lesions were the most common oral mucosal infectious diseases that are more prevalent amongst females and middle aged people.
